# Coupling Phase Behavior of Fatty Acid Containing Membranes to Membrane Bio-Mechanics

**DOI:** 10.3389/fcell.2019.00187

**Published:** 2019-09-19

**Authors:** Arwen I. I. Tyler, Jake L. Greenfield, John M. Seddon, Nicholas J. Brooks, Sowmya Purushothaman

**Affiliations:** ^1^Department of Chemistry, Imperial College London, London, United Kingdom; ^2^School of Food Science and Nutrition, University of Leeds, Leeds, United Kingdom; ^3^Department of Chemistry, University of Cambridge, Cambridge, United Kingdom; ^4^Department of Material Science, University of California, Davis, Davis, CA, United States; ^5^Cavendish Laboratory, Cambridge, United Kingdom

**Keywords:** *cis*, *trans*, lipid, membrane, bending rigidity, diabetes, polyunsaturated fatty acid

## Abstract

Biological membranes constantly modulate their fluidity for proper functioning of the cell. Modulation of membrane properties via regulation of fatty acid composition has gained a renewed interest owing to its relevance in endocytosis, endoplasmic reticulum membrane homeostasis, and adaptation mechanisms in the deep sea. Endowed with significant degrees of freedom, the presence of free fatty acids can alter the curvature of membranes which in turn can alter the response of curvature sensing proteins, thus defining adaptive ways to reconfigure membranes. Most significantly, recent experiments demonstrated that polyunsaturated lipids facilitate membrane bending and fission by endocytic proteins – the first step in the biogenesis of synaptic vesicles. Despite the vital roles of fatty acids, a systematic study relating the interactions between fatty acids and membrane and the consequent effect on the bio-mechanics of membranes under the influence of fatty acids has been sparse. Of specific interest is the vast disparity in the properties of *cis* and *trans* fatty acids, that only differ in the orientation of the double bond and yet have entirely unique and opposing chemical properties. Here we demonstrate a combined X-ray diffraction and membrane fluctuation analysis method to couple the structural properties to the biophysical properties of fatty acid-laden membranes to address current gaps in our understanding. By systematically doping pure dioleoyl phosphatidylcholine (DOPC) membranes with *cis* fatty acid and *trans* fatty acid we demonstrate that the presence of fatty acids doesn’t always fluidize the membrane. Rather, an intricate balance between the curvature, molecular interactions, as well as the amount of specific fatty acid dictates the fluidity of membranes. Lower concentrations are dominated by the nature of interactions between the phospholipid and the fatty acids. *Trans* fatty acid increases the rigidity while decreasing the area per lipid similar to the properties depicted by the addition of saturated fatty acids to lipidic membranes. *Cis* fatty acid however displays the accepted view of having a fluidizing effect at small concentrations. At higher concentrations curvature frustration dominates, leading to increased rigidity irrespective of the type of fatty acid. These results are consistent with theoretical predictions as detailed in the manuscript.

## Introduction

Biomembranes are composed of a wide variety of lipids, fatty acids, proteins, and cholesterol that play a crucial role in membrane-mediated processes. The composition of membranes is actively regulated to maintain membrane fluidity, structure, viscosity, and stress (Funari et al., 2003). Modulation of these biophysical properties can be monitored to understand membrane-associated processes like protein–lipid interactions, enzymatic activity, and regulation of surface receptors (Klausner et al., 1980).

One class of biomolecule that crucially affects membrane fluidity is fatty acids. Occurring as mono- or poly-unsaturated chains, fatty acids play a crucial role in various cellular processes (DeLong and Yayanos, 1985; Shaikh and Edidin, 2006; Mota et al., 2013; Picard and Daniel, 2013; Turk and Chapkin, 2013). For example, in neurodegenerative disorders like Alzheimer’s disease and Huntington disease, disruptions in fatty acid biosynthesis have been closely associated with the progression of the disease. One theory suggests that Sterol Regulatory Element Binding protein (SREBP) regulates lipid homeostasis by sensing the level of cholesterol in the cell, and provides negative feedback in synthesizing more cholesterol. Upon activation, SREBP acts as a transcription factor and stimulates expression of enzymes that regulate the fatty acid biosynthesis pathway (Sameni et al., 2018). The reduced biosynthesis of cholesterol and fatty acids is one of the early events in Huntington disease. Similar roles of perturbation of membrane fluidity upon addition of fatty acids have been reported recently. Piezo1, a mechano-sensitive channel, regulates crucial cellular processes like vascular architecture, cell migration, and erythrocyte volume. A study revealed that membrane fluidity was sensitive to the type of fatty acids incorporated, which in turn affected the activation of Piezo1 (Romero et al., 2019). Understanding how fatty acids affect membrane fluidity thus poses a biologically relevant challenge. Coupling the bio-mechanical properties of membranes with structural reorganization taking place as a result of addition of biomolecules could act as a powerful bio-marker in identifying the onset of disease. One of the advantages of using membrane mechanics as a biomarker lies in delineating the need to map complex, less known biochemical events that alter membrane function.

**FIGURE 1:**
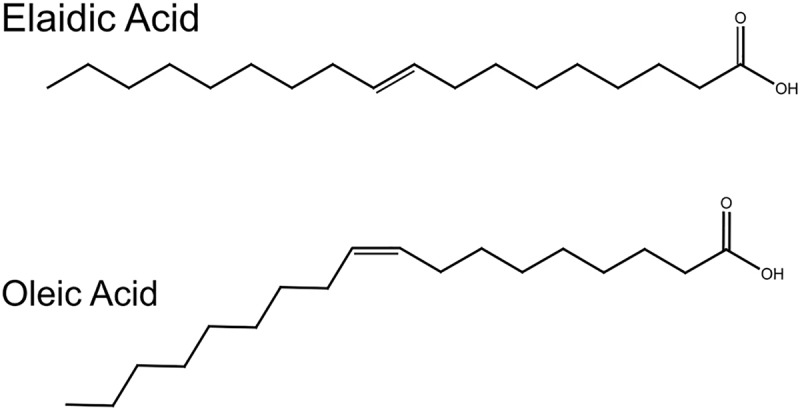
Structures of oleic and elaidic acids. Oleic acid and elaidic acids are congeners with a double bond at the 9th carbon position. Oleic acid has a kink due to the *cis* double bond.

Motivated by developing a complete structure – mechanics study of the effect of fatty acids on lipid membranes, we combined X-ray diffraction measurements (*structural information*) with bending energy measurements (*Bio-mechanical information*) on phospholipid membranes doped with fatty acids. As a proof-of-concept, we demonstrate the effect of two mono-unsaturated fatty acids, oleic acid (OA) and elaidic acid (EA) differing in the *cis*–*trans* configuration of the double bond (Figure 1), on dioleoylphosphatidylcholine (DOPC) bilayers. Both OA and EA have same molecular weight but the presence of a kink in OA makes them structurally different; EA has a more cylindrical shape and resembles saturated fatty acids. Here, we sought to understand how molecular conformations of EA and OA effect lipid bilayer membrane structure and mechanics.

Both EA (*trans*) and OA (*cis*) have been shown to be associated with a variety of health conditions like coronary heart disease, atherosclerosis, Alzheimer’s disease, diabetes, and cancer (Yang et al., 2011; Yaghmur et al., 2012; Turk and Chapkin, 2013). In the case of type 2 diabetes, it was reported that presence of *cis* fatty acids changes membrane flexibility which in turn causes a disruption in glucose transporter protein activity (Weijers, 2016). OA has also been associated with disruption of secretion of growth hormones associated with obesity by affecting the functioning of membrane proteins and proton flux across bilayers (Martin−Moreno et al., 1994; Hardman, 2004; Menendez et al., 2006; López-Miranda et al., 2010).

Given the importance of free fatty acids in cellular function, various biophysical studies have been carried out using artificial minimal systems. For example, structural changes in the bilayer membrane upon addition of fatty acids have been performed using X-ray diffraction and NMR techniques (Jilausner et al., 1980; Seddon et al., 1997; Templer et al., 1998; Inoue et al., 2001; Patel et al., 2001; Funari et al., 2003; Roach et al., 2004; Booth, 2005; Árnadóttir and Chalfie, 2010; Soubias et al., 2010; Yang et al., 2011; Vanni et al., 2014; Shaikh et al., 2015). X-ray diffraction studies by Seddon et al. (1997, 2006) revealed that addition of fatty acids influences membrane gel to L-alpha phase transitions and alters the lateral stress profile across the monolayer, favoring curved hexagonal and/or cubic phases. Upon addition of fatty acids to PC membranes the hydrophilic/hydrophobic balance is altered. This in turn affects the headgroup area to volume ratio which induces a curvature stress causing the transition to non-lamellar phases. In other studies (Mareš et al., 2008; Perutkova et al., 2009; Perutková et al., 2011), a closer look at the pivotal plane radius as a function of chain stiffness and internal curvature revealed that the increase in chain stiffness and internal curvature leads to a reduced pivotal plane radius favoring hexagonal phase transitions.

To obtain both structural as well as mechanical properties of bilayer membranes in response to the addition of EA and OA, we combined (a) vesicle fluctuation analysis on giant unilamellar vesicles (GUVs) for extracting the bending rigidity using our in-house algorithm, and (b) performed X-ray diffraction studies on simple DOPC/EA and DOPC/FA lipidic systems. Our results demonstrate that both EA and OA increase the bending rigidity of the membrane. Structural studies using X-ray diffraction on fatty acid containing membranes show a topological transition to non-lamellar inverse hexagonal structures in both EA and OA containing membranes.

Based on the previous models described above, we expect an increase in the bending rigidity of GUVs upon addition of fatty acids owing to the increase in curvature frustration. Systematic addition of EA and OA to DOPC membranes, however, induced different effects on the bending rigidities, with EA containing DOPC vesicles showing a smaller increase compared to the OA containing vesicles. Interestingly, these results are consistent with theoretical predictions, as discussed below.

## Experimental

### Materials and Methods

1,2-Dioleoyl-*sn*-glycero-3-phosphocholine (DOPC), OA, and EA were bought from Sigma–Aldrich, Inc. (Gillingham, United Kingdom). The lipids had a purity of >99% and were used without further purification.

### Sample Preparation for SAXS Measurements

Each lipid was freeze-dried individually, and samples were prepared by either mixing the desired amount of dry or stock solutions of lipids or dissolving them in chloroform. They were subsequently vortexed, dried under a steam of N_2_ gas, and lyophilized for a minimum of 24 h. Samples were hydrated with 70 wt% HPLC grade water (VWR, United Kingdom) and subjected to a minimum of five freeze–thaw cycles in order to achieve reproducibility and sample homogeneity. Due to the lack of ions in the HPLC water, its pH couldn’t be tested accurately using a pH meter but when tested with a pH universal indicator strip (Merck, Germany) it was shown to lie between 5 and 6. For the pH-dependent studies the pH of HPLC grade water was adjusted using HCl and NaOH to give a value of 3, 4, 5, 6, and 7 (±0.1) at 25°C as judged by a pH meter and universal indicator strips.

### Preparation of GUVs

Bending rigidity measurements were performed on GUVs that were produced using the electroformation method (Miglena and Dimitrov, 1986; Angelova et al., 1992). Briefly, lipid mixtures were dissolved in 9:1 CHCl_3_:CH_3_OH solution at a concentration of 0.8 mg/ml. Two microliter drops of the solution were spread on ITO-coated glass plates and dried in a lyophilizer for 30 min. A 1 mm thick PDMS spacer was sandwiched between the two ITO plates to hold the solution used to hydrate the sample. The well was then filled with 100 mM sucrose solution. An AC voltage of 2.6 V at 10 Hz was applied to the plates for 3 h. The voltage was then increased to 4.6 V and the frequency reduced to 4.4 Hz for 15 min. The temperature was set so that the lipids were always below their chain melting temperature (24°C). GUVs were in the size range of 10–50 μm.

Giant unilamellar vesicles were re-suspended in 125 mM glucose solution. A visualization chamber was made using a 50 × 25 mm glass coverslip and an acrylic well. Around 80 μl of glucose solution was added into the well. Twenty microliters of GUVs were suspended into the glucose solution. The chamber was sealed with a cover slip to avoid air currents and reduce stray light due to scattering. The relaxation time scales of these fluctuations are in the range of a few milliseconds to a few seconds. The relaxation time decreases as the cubic power of the mode number. For example, the relaxation time scale τ_*m*_ is given by τ_*m*_ ∼4η*R*^3^/κ*_*c*_*m^3^ where τ_*m*_ is the relaxation time for a given mode m, η is the viscosity of the medium, *R* is the radius of the vesicle, and κ*_*c*_* the bending rigidity (Henriksen et al., 2004). This means that higher modes have shorter characteristic time scales. For video recording, a fast CMOS camera from Infinity (Lumenera, INFINITY 1-2 2.0 megapixel) was used. In order to capture the fluctuations, the camera integration time (exposure time) should be as short as possible. The exposure was kept to 1 ms and videos of approximately 1 min in time were recorded. All measurements were taken at 24°C.

### Bending Rigidity

**FIGURE 2:**
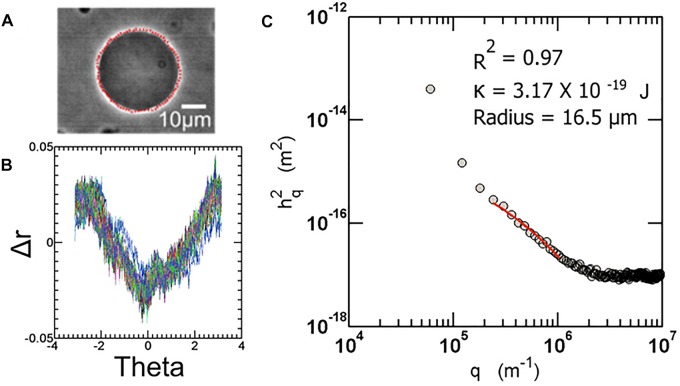
**(A)** Sample image of a fluctuating vesicle at 23°C **(B)** The contour extracted is converted to *r*-theta which is further Fourier transformed to obtain a power spectrum as shown in **(C)**. All the automation routines are built in-house using LabVIEW software.

Vesicle fluctuation analysis (VFA) was used to extract the bending rigidity of the quasi spherical fluctuating vesicles (Figure 2). Briefly, the edges of the fluctuating vesicles were extracted using a fully automated LABVIEW routine based around the maximum intensity fitting of the bright edges. For a symmetric composition membrane, the spontaneous curvature vanishes. For small deformations in a membrane, the deviations from the mean shape are Fourier transformed and using the equipartition theorem, the mean square amplitude of each mode (*q*_*x*_) is given by:

(1) $$\left\langle h{\left(q_{x},y=0\right)}^{2}\right\rangle =\frac{1}{L}\frac{k_{\text{B}}\,T}{2\,\text{σ}}\left[\frac{1}{q_{x}}-\frac{1}{\sqrt{\frac{\text{σ}}{{\text{κ}}_{c}}+q_{x}^{2}}}\right]$$

where *k*_*B*_ is the Boltzmann constant, *T* is the temperature, σ is the membrane tension, κ*_*c*_* is the bending rigidity, and *L* is the average circumference of the vesicle contours taken over all frames.

For bending dominated fluctuations, where $\frac{\sigma }{\left(\frac{q_{x}^{2}}{{\text{κ}}_{c}}\right)}\to 0$

We have

(2) $$\left\langle h{\left(q_{x},y=0\right)}^{2}\right\rangle =\frac{1}{4\,L}\frac{k_{\text{B}}\,T}{{\text{κ}}_{c}\,q^{3}}$$

where *L* = 2π<*r*> is the average circumference of the contour of all frames. Experimentally, it is required to calculate the Fourier transformation of the fluctuations about the mean radius of vesicle and relate it to the continuous Fourier transform *h*(*q*). After finding the co-ordinates of the contour and representing them in polar coordinates, the mean radius of the vesicle <*r*> is calculated for all *r*(θ*_*n*_*), where *n* is the number of points on the circumference per frame, over all frames. The amplitude of fluctuation about the mean radius is given by *h*_*m*_ = *r*(θ*_*n*_*)−<*r*>. The FFT of the fluctuations is performed by using an inbuilt LabVIEW function.

We consider only in-plane fluctuations since only these fluctuations can be captured with the microscope. Different modes depict regimes of the power spectrum governed by different parameters. The lower modes are mainly dominated by tension, or displacement factors of the vesicle. The intermediate regime (approx. between modes 6 and 20) represents the bending dominated region. This region is fit to the above equation to extract the bending rigidity. Detailed description of the method can be obtained from many excellent articles on the subject (Pécréaux et al., 2004; Yoon et al., 2010). The fluctuation analysis technique used here was developed in-house, and is described in depth in previous articles (see the Supplementary Information of Henriksen et al., 2004) (Elani et al., 2015; Purushothaman et al., 2015).

### SAXS Measurements

X-ray experiments were carried out using a custom-built small angle X-ray beamline having a microsource X-ray generator (Bede Ltd., Durham, United Kingdom) producing X-rays with λ = 1.54 Å. A low divergence (2 mrad) X-ray beam is generated by monolithic poly-capillary optics (X-ray Optical Systems, Inc., United States). Diffraction patterns were recorded on a Gemstar intensified CCD X-ray detector (Photonic Science Ltd., Battle, United Kingdom). Sample capillaries were mounted in a custom-designed copper sample holder with Peltier temperature control (Melcor, United States), having an accuracy of ±0.1°C. The sample to detector distance was set to 200 mm, giving an accessible *q* range between 0.39 and 0.048 Å^–1^. Samples were allowed to equilibrate for 10 min before each diffraction pattern was recorded.

Silver behenate (layer spacing *d* = 58.38 Å) was used to calibrate the low-angle X-ray diffraction data for all measurements. Diffraction images were analyzed using the IDL-based AXcess software package, developed in-house by Dr. A. Heron (Seddon et al., 2006). All the experiments were performed on three independent samples for each composition.

## Results and Discussion

We first performed small angle X-ray diffraction measurements to investigate the effect of compositional variation of fatty acids in DOPC membranes. The phase behavior of DOPC membranes with varying composition of fatty acids is presented below. Next, we describe the biomechanics of membranes containing *cis* and *trans* free fatty acids by extracting the bending rigidity of GUVs, using the fluctuation analysis developed in-house.

### Phase Behavior

**FIGURE 3:**
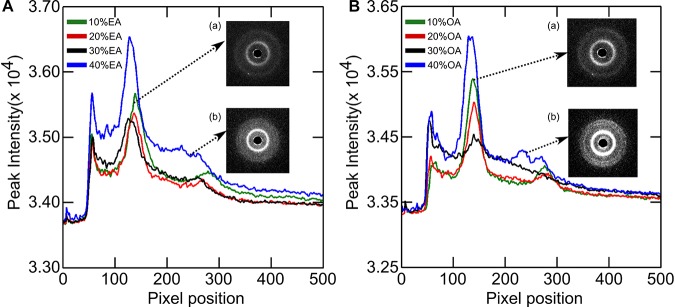
X-ray diffraction images of **(A)** DOPC + Elaidic acid (EA) and **(B)** DOPC + Oleic acid (OA) mixtures. As the amount of fatty acids increase, peaks corresponding to inverse hexagonal phase starts appearing. See inset (b). Inset (a) represents the initial lamellar peaks.

The phase behavior of DOPC:OA mixtures at limited hydration has recently been explored (Gillams et al., 2014). Here we investigate the phase behavior of DOPC:OA and DOPC:EA mixtures of up to 40 mol% fatty acid in excess (HPLC water, pH ∼5.5) water (70 wt%) at 25°C. DOPC:OA mixtures in HPLC water containing up to 20 mol% OA adopt a lamellar phase which slightly shrinks in layer spacing as the concentration of OA increases (Figure 3). At 30 mol% OA the lamellar phase is seen to coexist with a small broad hump under the first- and second-order reflections, and its lattice parameter is shifted to higher values (Gillams et al., 2014). Previous studies have shown that DOPC:OA mixtures at limited hydration form a lamellar phase coexisting with swollen Pn3m and Im3m cubic phases at 20 mol% OA. They also observed a hexagonal (H_*II*_) phase coexisting with the swollen cubic phases at 30 mol% OA (Gillams et al., 2014). Due to the accessible *q*-range in our set-up we cannot determine whether the lamellar phase at 30 mol% OA is coexisting with swollen cubic phases. However, the similarity in phase behavior between the two studies indicates that it might be possible to attribute the broad hump to higher order reflections of swollen cubic phases, which are unresolved. At 40 mol% OA, the system transforms to a pure H_*II*_ phase with a layer spacing of 74.6 ± 0.05 Å.

**FIGURE 4:**
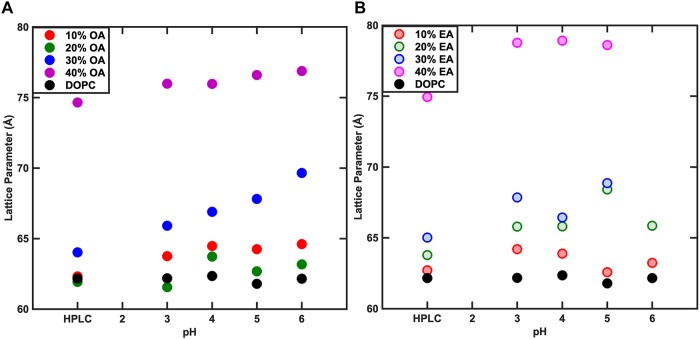
Effect of pH on the phase behavior of fatty acid containing membranes. As the concentration of Fatty acid is changed, the lattice parameter changes however, the variation in the lattice parameter with pH is negligible. At 40% Fatty acid, both Oleic acid (OA) **(A)** and Elaidic acid (EA) **(B)** containing membrane systems adopt an H_*II*_ phase.

The phase behavior of DOPC:EA mixtures is similar. Between 10 and 30 mol% EA a pure lamellar phase is formed which transforms to a pure H_*II*_ phase at 40 mol% EA with a layer spacing of 74.9 Å (Figure 4). Here, the lattice parameter of the lamellar phase is seen to increase with increased EA concentration.

**FIGURE 5:**
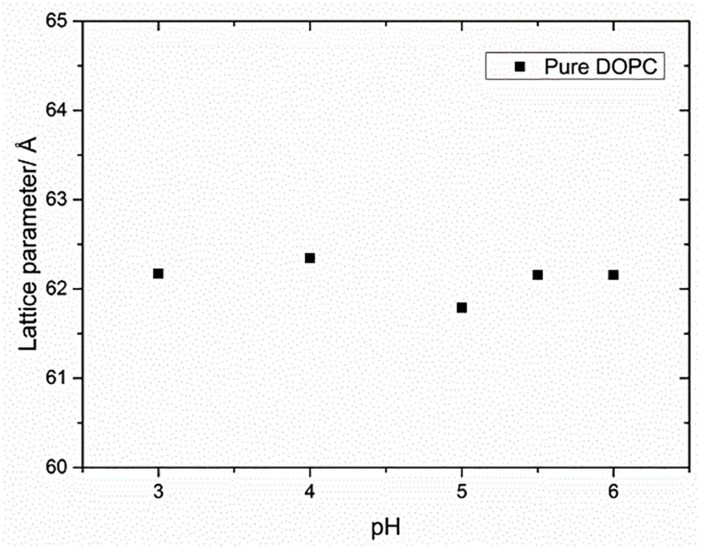
Effect of pH on the lattice parameter of pure DOPC membranes. The lattice parameter remained constant for the range of pH values indicating that the effect of pH on lipid mixtures was negligible.

The pK_*a*_ of OA and EA has been reported to vary largely, with values ranging from 4.8 to 9.95 (Kanicky and Shah, 2002; Salentinig et al., 2010; Bennett et al., 2013). The reason for this large discrepancy is that the value depends on whether the pK_*a*_ has been measured for the fatty acid as a monomer in solution, in a micellar solution, or incorporated in a lipid bilayer. The pH of the water used in this study was ∼5.5. To test whether the change in the lattice parameter of the fatty acid containing DOPC mixtures used here was due to the electrostatic interactions of the fatty acids at pH 5.5 or due to the molecular structure of the fatty acid molecules, the effect of pH (3–6) on the phase behavior and resulting structures was also investigated. Figure 4 shows that the phase behavior of the binary mixtures is unchanged with pH within this range. The layer spacing of the lamellar phase is relatively constant at different pH values for each mixture; however, the trend with increased fatty acid concentration is the same, indicating that the effect on the layer spacing is due to the molecular structure of the fatty acid molecules and not electrostatics, as the fatty acids should be fully protonated at pH 3. In order to confirm that the change in layer spacing is not related to protonation of the DOPC headgroup at low pH, the layer spacing of pure DOPC was also investigated between pH 3 and 6, and was found to be unchanged (Figure 5).

### Bending Rigidity

**FIGURE 6:**
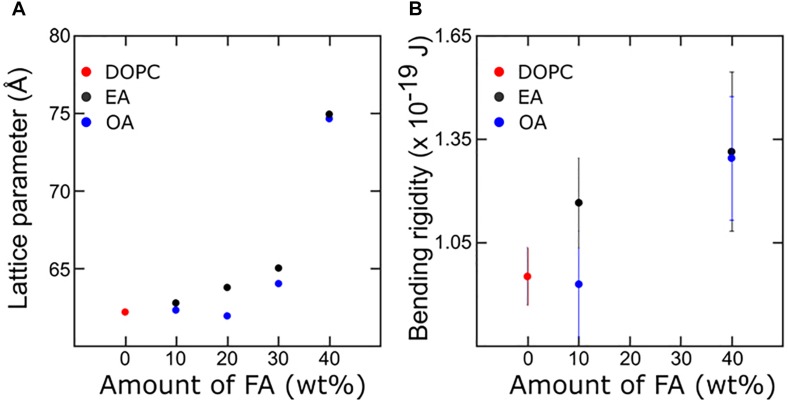
Combined X-ray diffraction **(A)** and bending rigidity plot **(B)** describing membrane mechanics with respect to the corresponding change in molecular arrangement. The bulk 40% Fatty acid samples are in the H_*II*_ phase whereas in the GUVs they are confined to a bilayer structure.

The effect of mono-unsaturated fatty acids on the bending rigidity of the lipid membrane was studied by capturing membrane fluctuations of GUVs made from DOPC and fatty acid mixtures as described. Figure 6 shows the bending rigidities of DOPC vesicles with varying amounts of OA and EA. The results are from the analysis of about 25–30 vesicles per sample. Addition of fatty acid to DOPC has different effects on the bending rigidity of the membrane depending on the fatty acid added. Addition of 10 mol% OA only marginally decreases the bending rigidity (9.2 × 10^–20^ ± 1.1 × 10^–2^ J) while 10 mol% EA increases it to 1.1 × 10^–19^ ± 1.3 × 10^–20^ J compared to the bending rigidity of pure DOPC vesicles (9.5 × 10^–20^ ± 0.8 × 10^–20^ J). At 40 mol% of fatty acid, however, the bending rigidity of both OA and EA containing DOPC vesicles shows a large increase compared to 10 mol% fatty acid mixtures. It should be noted that this increase is less pronounced for EA compared to OA.

We will discuss the information obtained from X-ray diffraction measurements and couple the corresponding effect in the membrane mechanics. The differences in the effect on bending rigidities between the two fatty acids on DOPC membranes can be attributed to a number of factors, such as the phase behavior and the molecular structure of the individual fatty acid molecules.

Considering the lateral stress profile of a DOPC bilayer, addition of fatty acids should reduce the headgroup pressure. Consequently, this should be compensated by an increase in chain pressure. It is known that addition of fatty acids to phospholipids increases the negative spontaneous curvature of the lipid monolayer by increasing the hydrocarbon chain pressure, and curvature frustration then favors the formation of inverse phases (Zimmerberg and Gawrisch, 2006). The presence of a *cis* double bond in OA increases the splay of the hydrophobic chains more than its *trans* counterpart EA. For a given composition the effect of the presence of OA in bilayer forming lipids like DOPC will be greater in comparison to EA/DOPC mixtures. As a result, a lower concentration of OA is required to form inverse phases compared to EA. The observed phase behavior of the DOPC-EA and DOPC-OA systems supports this prediction.

**FIGURE 7:**
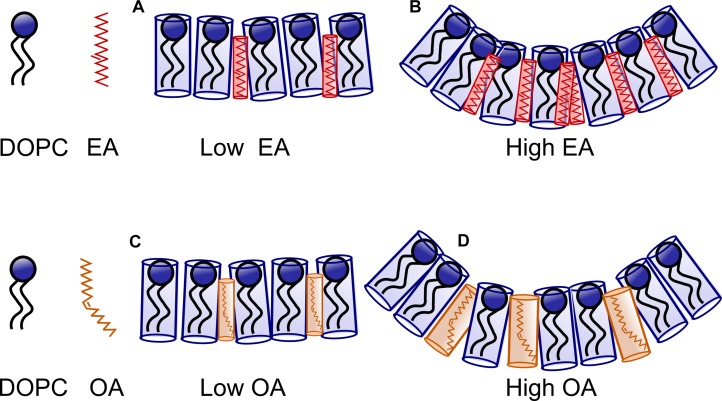
Schematic representing the effect of Oleic acid (OA) and Elaidic Acid (EA) on DOPC membranes. At low concentration of EA/FA **(A)**, the effect of FA on the bilayer is small. At higher concentration curvature frustration dominates as the FA remain incompressible. However, more EA **(B)** is required to drive the system to a non-lamellar phase in comparison to OA. At low concentrations, OA containing membranes are similar to EA containing membranes. However, at high concentrations **(C)**, relatively less OA is required to induce curvature **(D)** owing to the kink in the structure of OA.

Theoretical estimates made by Szleifer et al. (1990) on the dependence of bending rigidity on the average area per chain have shown that there is a dramatic decrease in bending rigidity as the area/molecule increases or when the curvature frustration is reduced, e.g., by addition of short chain amphiphiles (Figures 13, 14 of Salentinig et al., 2010). In our experiments, EA can be treated as a saturated amphiphile owing to its kinked structure at the double bond. Furthermore, studies (Leekumjorn et al., 2009) have shown that addition of *saturated* fatty acids to DOPC membranes decreases the average area per lipid linearly with concentration. On the contrary, addition of *unsaturated* fatty acids like OA has a less pronounced effect at low concentrations (up to 10 mol%). The area per lipid decreases less sharply upon further increase of *unsaturated* fatty acids. The average area per molecule of *saturated* fatty acid is low and unchanged upon increase in concentration in a DOPC bilayer. The area/molecule of *unsaturated* fatty acid like OA is higher and decreases as their concentration in the bilayer is increased. This has been attributed to the fact that the *saturated* fatty acid (EA) is relatively incompressible in the bilayer. The *unsaturated* fatty acids (OA), on the other hand, can order their chains by straightening their unsaturated chains. At high concentrations (>25 mol%) of fatty acid the area occupied by both saturated and unsaturated fatty acids is the same (Figure 7).

We look at the bending rigidities of vesicles containing fatty acid. Addition of 10% EA to DOPC membrane increases the lattice parameter which indicates a decrease in the area/lipid molecule (Leekumjorn et al., 2009). According to the theoretical predictions described above, a decrease in area/lipid causes an increase in the bending rigidity.

**FIGURE 8:**
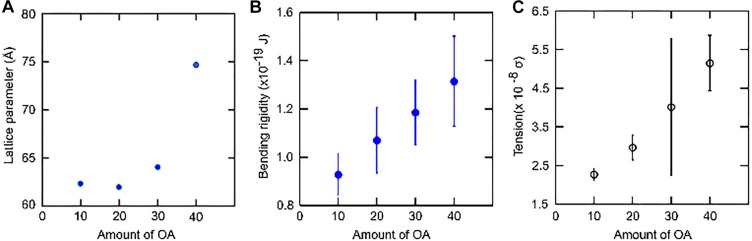
Effect of systematic increase in the concentration of Oleic acid (OA) on DOPC membranes. **(A)** As the concentration increases, the lattice spacing increases. **(B)** The bending rigidity increases as the area per lipid decreases. **(C)** The tension in the membrane also increases. However, VFA gives bending rigidity values more reliably in comparison to tension measurements.

On the other hand, the addition of 10 mol% OA has very little effect on the lattice parameter of the lamellar phase compared to pure DOPC membranes. The bending rigidity of the binary mixture is slightly lower. OA increases membrane fluidity by decreasing the packing between phospholipids. This leads to increased fluctuations in the bilayer (Calder et al., 1994; Leekumjorn et al., 2009) which is reflected in the lowering of bending rigidity (Figures 6, 8). One of the best ways to describe membrane fluidity is the lipid chain order parameter, which is a measure of the amplitude of the splay in the alkyl chains. An increase in order parameter indicates a decrease in chain conformational motion, tending to reduce membrane fluidity (Mills et al., 2008). The order parameter can be estimated using X-ray (Mills et al., 2008) diffraction measurements and NMR (Vameer et al., 2007) studies. It is expressed as:

$S=\frac{1}{2}\,\left(3\,\left\langle {\text{cos}}^{2}\,\text{β}\right\rangle -1\right)$, where *S* is the lipid chain average orientational order parameter and β is the average chain tilt angle from the bilayer normal.

At 20 mol% OA, there is a slight decrease in layer spacing at all pH measurements. Although a decrease in lattice parameter might imply a slight increase in area/molecule and hence a lower bending rigidity, we noted that the bending rigidity did not decrease, suggesting that the decrease in spacing is due to the water layers between the bilayers becoming slightly thinner.

At 30 mol% OA, however, the layer spacing of the lamellar phase increased. Consequently, the bending rigidity also rises consistent with the theoretical predictions.

As mentioned earlier, the lamellar peak at 30 mol% OA is seen to coexist with a broad hump under the first-order reflection. We suggest that the origin of the broad hump might be poorly resolved higher order reflections of a cubic phase as seen by Gillams et al. (2014). If the system has a propensity to form a non-bilayer structure but is forced to remain in a bilayer arrangement in a vesicle then the bending rigidity will rise as the curvature elastic stress increases.

Taken together, at low OA concentrations, we believe that the forces dominating the bending rigidity are due to the effect of the molecular interactions between the OA and DOPC membrane, namely the increased fluidity of the bilayer, in contrast to the less “floppy” EA. As the concentration of fatty acid is increased, there is a decrease in area per (lipid + fatty acid) due to ordering of the unsaturated chains. This leads to an increase in the bending rigidity.

At 40 mol% fatty acid, however, the dominant force influencing the bending rigidity is curvature frustration for both the OA and EA systems. It has been shown that above 25 mol% fatty acid in DOPC, the area occupied by both saturated and unsaturated fatty acids is the same. As a result, the difference in the area occupied by the DOPC:OA and DOPC:EA membranes is less pronounced. Both fatty acids will thus have a similar effect on bending rigidity at higher concentrations.

**FIGURE 9:**
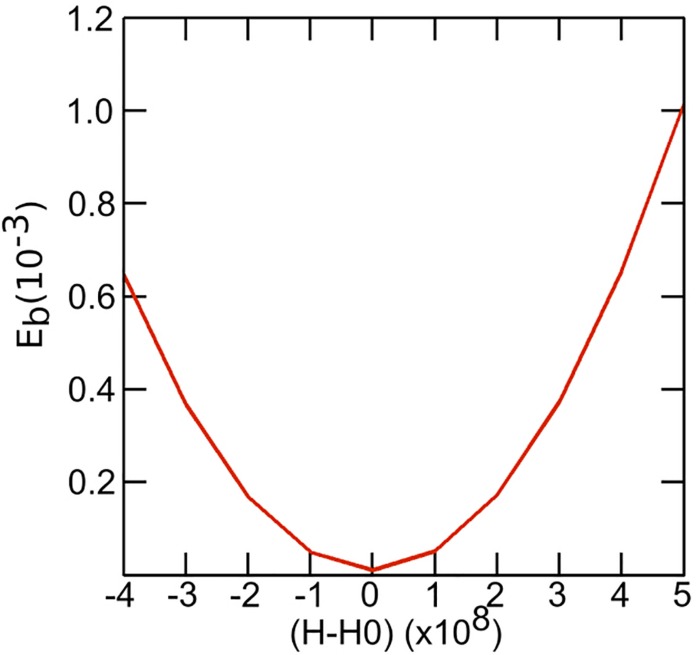
Plot depicting the variation of bending rigidity as a function of spontaneous curvature. For fluctuations upto 100 nm we can see that the bending rigidity rises sharply if the system deviates from its preferred spontaneous curvature (H_0_).

As seen by SAXS, bulk DOPC:fatty acid systems at 40 mol% fatty acid adopt an inverse hexagonal phase. However, within a GUV they are forced to be in a bilayer state which leads to a sharp increase in the curvature frustration and a pronounced increase in the bending rigidity. Assuming the lipids are evenly distributed within the two leaflets, we can approximately estimate the effect on bending rigidity as the system deviates (or is forced to deviate) from its preferred radii of curvature. The fluctuation analysis studies show that fluctuations are in the order of 100 nm. Using this value as an estimated radius of curvature we can see that the bending rigidity rises sharply if the system deviates from its preferred spontaneous curvature (H_0_) (Figure 9).

Mareš et al. (2008) detailed the relationship between intrinsic curvature and chain stiffness and the pivotal plane cross section radius to explain lamellar to hexagonal phase transitions. The model is based on anisotropic shape of lipid molecules described by principal intrinsic curvatures. The study demonstrated that as the chain stiffness and intrinsic curvature increase, the pivotal plane radius decrease leading to transition from lamellar to hexagonal phase. The decrease in the pivotal plane radius is thus a result of competition between the bending and interstitial energy. When the chain is stiff, there is propensity to curve toward the hexagonal corners to fill voids which leads to transition to hexagonal phase. This could explain the transition to inverted hexagonal phase at high fatty acid concentrations when the bending rigidity also increases.

## Conclusion

In conclusion, the effect of *cis-* and *trans-*monounsaturated fatty acid on bilayer mechanics is studied using both X-ray diffraction and vesicle fluctuation analysis. Addition of fatty acids increases the bilayer bending rigidity. EA with its *trans* double bond behaves more like a saturated amphiphile, showing less change in area/molecule, and causing a smaller change in bending rigidity. The presence of *cis* double bonds in OA increases the probability of inducing negative curvature, increasing curvature frustration, and an increase in bending rigidity. Furthermore, higher concentrations show a dominant effect of curvature frustration, leading to higher bending rigidity, whereas at lower concentrations of fatty acids, several intermolecular interactions as well as the floppiness of the fatty acids dictate the thickness and rigidity of membranes. The theoretical model developed by Szleifer et al. (1990) to explain the curvature of lipidic membranes was useful in interpreting some of the findings reported here. Our observations show that the bending rigidity is sensitive to the type of fatty acids incorporated into DOPC membranes. The study demonstrates the potential application of bio-mechanical properties as unique bio-markers to identify membrane reorganization as a result of disruption in fatty acid biosynthesis as seen in certain neurodegenerative conditions.

## Author Contributions

JG performed all the bending rigidity measurements. AT performed all the X-ray diffraction and pH measurements. SP lead the project. JS provided guidance and helped in preparing the manuscript. NB was part of the project grant and provided funding support.

## Conflict of Interest Statement

The authors declare that the research was conducted in the absence of any commercial or financial relationships that could be construed as a potential conflict of interest.
